# Credibility assessment of patient-specific computational modeling using patient-specific cardiac modeling as an exemplar

**DOI:** 10.1371/journal.pcbi.1010541

**Published:** 2022-10-10

**Authors:** Suran Galappaththige, Richard A. Gray, Caroline Mendonca Costa, Steven Niederer, Pras Pathmanathan

**Affiliations:** 1 Center for Devices and Radiological Health, US Food and Drug Administration, Silver Spring, Maryland, United States of America; 2 School of Biomedical Engineering & Imaging Sciences, King’s College London, London, United Kingdom; University of Michigan, UNITED STATES

## Abstract

Reliable and robust simulation of individual patients using patient-specific models (PSMs) is one of the next frontiers for modeling and simulation (M&S) in healthcare. PSMs, which form the basis of digital twins, can be employed as clinical tools to, for example, assess disease state, predict response to therapy, or optimize therapy. They may also be used to construct virtual cohorts of patients, for *in silico* evaluation of medical product safety and/or performance. Methods and frameworks have recently been proposed for evaluating the credibility of M&S in healthcare applications. However, such efforts have generally been motivated by models of medical devices or generic patient models; how best to evaluate the credibility of PSMs has largely been unexplored. The aim of this paper is to understand and demonstrate the credibility assessment process for PSMs using patient-specific cardiac electrophysiological (EP) modeling as an exemplar. We first review approaches used to generate cardiac PSMs and consider how verification, validation, and uncertainty quantification (VVUQ) apply to cardiac PSMs. Next, we execute two simulation studies using a publicly available virtual cohort of 24 patient-specific ventricular models, the first a multi-patient verification study, the second investigating the impact of uncertainty in personalized and non-personalized inputs in a virtual cohort. We then use the findings from our analyses to identify how important characteristics of PSMs can be considered when assessing credibility with the approach of the ASME V&V40 Standard, accounting for PSM concepts such as inter- and intra-user variability, multi-patient and “every-patient” error estimation, uncertainty quantification in personalized vs non-personalized inputs, clinical validation, and others. The results of this paper will be useful to developers of cardiac and other medical image based PSMs, when assessing PSM credibility.

## Author summary

Patient-specific models are computational models that have been personalized using data from a patient. After decades of research, recent computational, data science and healthcare advances have opened the door to the fulfilment of the enormous potential of such models, from truly personalized medicine to efficient and cost-effective testing of new medical products. However, reliability (credibility) of patient-specific models is key to their success, and there are currently no general guidelines for evaluating credibility of patient-specific models. Here, we consider how frameworks and model evaluation activities that have been developed for generic (not patient-specific) computational models, can be extended to patient specific models. We achieve this through a detailed analysis of the activities required to evaluate cardiac electrophysiological models, chosen as an exemplar field due to its maturity and the complexity of such models. This is the first paper on the topic of reliability of patient-specific models and will help pave the way to reliable and trusted patient-specific modeling across healthcare applications.

## 1. Introduction

Patient-specific computational models (PSMs) are computational human models that have been personalized to specific patients, rather than representing a generic, synthetic, or average patient. For example, over the course of many decades the field of computational heart modeling has transitioned from modeling electrical activity in single cells only, to modeling propagation of electrical waves in slabs of tissue, and then modeling the whole organ. Previously, generating a new model of a unique individual was a very time-consuming process. Now, it is possible to generate a personalized model from clinical imaging data rapidly and automatically, and then simulate the patient’s heart activity, all over the course of a few hours. Patient-specific modeling has also reached maturity in the field of fractional flow reserve estimation, for example software devices that generate personalized CT image-based models of coronary flow for functional evaluation of coronary arterial disease have been cleared in the US market [1]. Other applications of PSMs with devices on the market include preoperational planning and sizing for neurovascular surgery [2], and non-invasive mapping of heart surface electrical activity [3]; for more information see [4]. The technological advances that underly these devices and others have opened the door for patient-specific models being used as standalone medical device software, within medical devices, or as tools for evaluating medical products. Patient-specific modeling also form the basis of digital twins. In healthcare applications, the term ‘digital twin’ is sometimes used synonymously with patient-specific model. Alternatively, it has been defined more precisely as a “comprehensive, virtual tool that integrates coherently and dynamically the clinical data acquired over time for an individual using mechanistic and statistical models” [5]. The potential healthcare benefits of PSMs across all of these applications is enormous. However, using PSMs in safety-critical applications requires careful evaluation of model *credibility*–defined as the trust, based on available evidence in the predictive capability of a computational model [6].

Credibility of computational models for medical products has been the subject of considerable recent interest for the medical device industry. Credibility assessment involves several activities including verification (the process of determining if a computational model is an accurate implementation of the underlying mathematical model), validation (the process of determining the extent to which a computational model is an accurate representation of the real-world system that is being modeled) and uncertainty quantification (UQ; the process of characterizing uncertainties in the model, e.g., in model parameter values due to measurement error or population variability, and then computing the resultant uncertainty in model outputs). Verification can be broken down into code verification, which tests for potential software bugs, and calculation verification, which estimates numerical errors due to spatial or temporal discretization. A milestone event was the publication of the ASME V&V40 Standard in 2018 [6], which was the culmination of a multi-year collaboration involving modeling experts across the medical device industry and the Center for Devices and Radiological Health (CDRH) at the US Food and Drug Administration (FDA). This Standard provides a risk-based framework for evaluating the credibility of a computational model across device applications and was the first (and remains the only) such Standard in the medical product space. Briefly, the workflow in V&V40 is as follows. First, there are three preliminary steps: (i) defining the ‘question of interest’, that is, the specific question about the real world (e.g., regarding the medical device or a patient) that a model will be used to address; (ii) defining the ‘context of use’ (COU), that is, how exactly the model will be used to address the question of interest, and (iii) performing a risk assessment to characterize the risk to patients in using the model to address the question of interest. V&V40 then defines a number of ‘credibility factors’, which are factors to be considered when planning verification, validation and uncertainty quantification (VVUQ) activities. The list of categories of credibility factors is:

- Code verification credibility factors: software quality assurance and numerical code verification.
- Calculation verification credibility factors: discretization error, numerical solver error and use error.
- Validation credibility factors regarding the model: model form and model inputs (both broken down in sub-factors).
- Validation credibility factors regarding the comparator (i.e., the real-world data the model is compared to): test samples and test conditions (both broken down in sub-factors).
- Validation credibility factors regarding the comparison process: equivalency of inputs and output comparison (latter broken down in sub-factors).
- Applicability credibility factors which assess the relevance of the validation results to the COU: relevance of the quantities of interest and relevance of the validation activities to the COU.

For each credibility factor, V&V40 describes how users should define a ‘gradation’ of activities of increasing level of investigation. For example, for the ‘*software quality assurance*’ (SQA) credibility factor, a simple gradation is: (a) no SQA performed; (b) unit testing performed; (c) full SQA adhering to SQA Standards. V&V40 provides example gradations for each credibility factor. After defining a gradation, V&V40 describes how users should select a target level from the gradation based on the risk assessment. See (6) for full details.

A wide range of medical device models were considered during the development of V&V40; however, while it is clear how to apply the preliminary steps of credibility assessment approaches as in V&V40 regarding the question of interest, risk and COU to PSMs, it may not be clear what the unique characteristics of PSMs are when performing credibility assessments and how they can be considered in the subsequent stages of V&V40. In fact, how best to evaluate the credibility of PSMs has largely been unexplored; we are not aware of any article in the literature that identifies and discusses the unique considerations that arise when evaluating PSMs. The aim of this paper is to understand the unique considerations of credibility assessment of medical image based PSMs. By medical image based PSMs, we refer to a class of commonly developed PSMs that use medical imaging data (and potentially other patient data) as input, generate a patient-specific geometry and simulate a physical or physiological process on that geometry, typically using partial differential equations. For the remainder of this document, ‘PSMs’ refers to medical imaged based PSMs. The following applications are in scope: PSMs developed as software tools that can be applied to any new patient, and PSMs developed to create a ‘virtual cohort’ of patients for *in silico* medical device testing. However, virtual cohorts that have been extended by generating new ‘synthetic patients’ (e.g., by varying parameter values within ranges observed in the real patient population) are not within the scope of this paper, since in this case the synthetic patients do not correspond to any real patient (i.e., are not technically PSMs).

We use cardiac electrophysiological (EP) modeling as an exemplar. Cardiac EP modeling is a mature field with applications in clinical tools, medical device evaluation and drug safety evaluation, that requires processing of disparate sources of data and solving complex multiscale models. We believe that many of the challenges/nuance in assessing other medical image based PSMs will arise for cardiac PSMs, justifying using this field as an exemplar.

**Fig 1:**
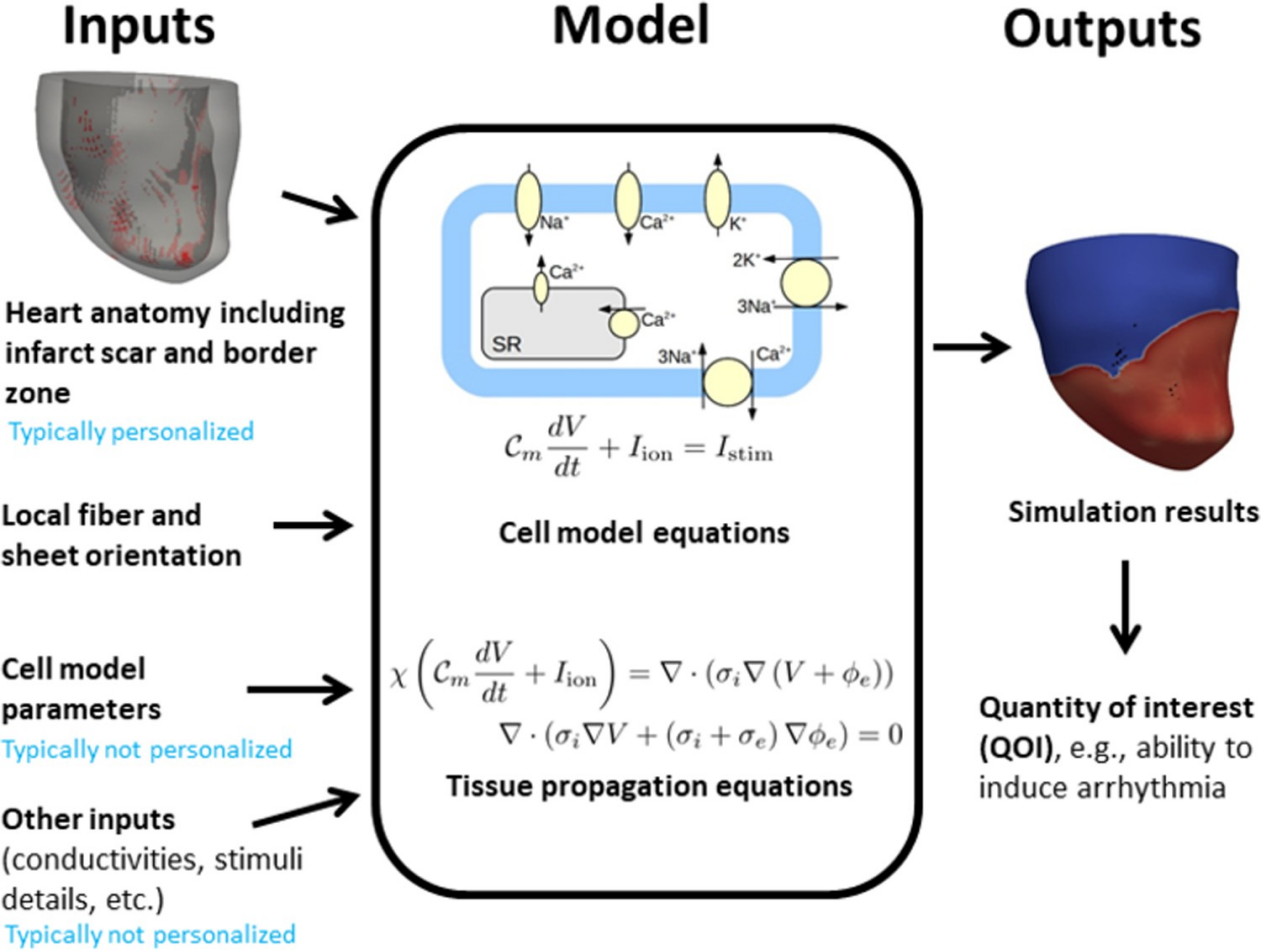
Overview of cardiac electrophysiological models.

Fig 1 illustrates the components of a model of cardiac EP. A fundamental component is the cell model, which is generally a system of ordinary differential equations that predict the time course of the transmembrane voltage (the action potential) and other cellular quantities, sometimes in response to external electrical stimuli (i.e., pacing or defibrillation). Many cell models have been developed, differing in which sub-cellular processes are included [7]. Cell models may have dozens of state variables and hundreds of parameters. To simulate electrical activity in tissue, the cell model is coupled to partial differential equations which govern electrical propagation in excitable tissue, typically the monodomain or bidomain equations [8]. To simulate electrical activity in the organ (atria, ventricles, or entire heart), the following are usually specified. (i) A computational mesh of the anatomy, typically generated from imaging data. (ii) Regions of non-excitable infarct scar, also determined from imaging. (iii) Regions of border zone (BZ) tissue. These are transient regions between scar and healthy tissue that are excitable but have different properties to healthy tissue. (iv) Fiber and sheet directions. These are orthogonal vector fields on the geometry indicating principal directions of conductivity. (v) Tissue conductivities in the fiber, sheet and normal-to-sheet directions. (vi) A stimulus protocol, such as apical pacing, pacing to replicate normal sinus rhythm, pacing at cardiac resynchronization therapy lead locations, etc. Other factors such as regional heterogeneities may also be included in the model. Previously, we considered VVUQ of generic cardiac models [9–12]; here we extend these works to cardiac PSMs.

We begin in Section 2 by reviewing cardiac EP PSMs, identifying modeling options that are unique to PSM but not applicable to generic models. We then consider how VVUQ applies to PSMs. In Section 3 we perform two simulation studies motivated by the results of Section 2 using a virtual cohort of 24 models incorporating patient-specific left ventricular geometry. The first is related to verification and assesses if discretization error varies across patients, and the second involves investigating how uncertainty in personalized and non-personalized inputs impacts conclusions of a virtual cohort simulation study. The survey and study results are then used in Section 4 to identify how important characteristics of PSMs can be considered when assessing credibility with the approach of the ASME V&V40 Standard.

## 2. Review of cardiac electrophysiological PSMs and VVUQ applied to PSMs

In this section we review approaches from previous publications used for developing cardiac EP PSMs, considering different aspects of cardiac models in turn. We focus our attention on modeling options that are unique to PSM but not applicable to generic models. We then discuss how VVUQ applies to PSMs.

### 2.1 Review of cardiac electrophysiological PSMs

While there are a wide range of possible **contexts of uses** for PSMs, most PSMs developed in the cardiac EP literature fall into one of the following two categories, which, as we shall see, it will be critical to distinguish between:

1. *PSMs as a* *clinical tool* *(PSM-CT)*. Here, the goal is to develop a clinical tool involving a PSM workflow, that can be applied to any new patient in the patient population using their own clinical data. Examples include PSMs for guiding cardiac ablation [13,14], risk stratification [15,16], cardiac resynchronization therapy (CRT) lead placement [17] and non-invasive mapping [18].
2. *A simulation study using a* *virtual cohort* *of PSMs (PSM-VC)*. Here, the goal is to investigate a question of interest about, for example, cardiac function or a drug/device, using a virtual cohort of PSMs that has been generated from data from several real patients. Examples include investigating ventricular fibrillation (VF) mechanisms [19], CRT efficacy [20], implantable cardioverter defibrillator efficacy [21], or to understand mechanisms related to CRT safety [22]

A key difference between these applications is that for clinical tools, the COU simulations require applying the full PSM workflow to a new patient, whereas for virtual cohorts the COU simulations will use the virtual patients that have already been constructed. For PSM-CTs, predictions are made about that specific patient and individual-level errors are potentially very consequential, whereas for some PSM-VCs individual-level errors may not impact the overall study conclusion (though this depends on what specifically the virtual cohort is used for–if the cohort was used to identify worst-case conditions individual-level errors may be very consequential). These categories are not intended to cover all PSM applications, and there are other PSM applications that fall outside these categories, for example, a simulation study using a single PSM or a clinical tool which use a virtual cohort. However, PSM-CTs and PSM-VCs as defined above cover the majority of PSM use-cases.

Choosing the **governing equations for tissue-level propagation** is one of the most important decisions that must be made for cardiac EP PSMs. Some options include the monodomain or bidomain [8], pseudo-bidomain [23], Eikonal [24] or reaction-Eikonal [25] formulations. PSMs often use simpler formulations due to speed constraints. For example, cardiac PSMs often use the Eikonal (e.g., [26]), reaction Eikonal (e.g. [22]) or monodomain (e.g., [13]) formulations.

The **level of personalization** is a fundamental choice that needs to be made when developing a PSM workflow. In general, any or all model inputs could be personalized. However, it is generally impractical for all inputs to be personalized in a physiological model; instead, there are typically many inputs that are fixed to generic values (nominally population average values). The decision on whether to personalize an input involves considering the COU, the disease state, importance of personalizing that input to obtain accurate patient-specific predictions, and practical considerations, i.e., ability to obtain patient-specific data. Typically, heart anatomy and scar/BZ regions (if modeled) are personalized, fiber directions are ‘semi-personalized’, whereas conductivities and cell model parameters are less often personalized. These are discussed further below.

The **heart geometry** is routinely personalized in cardiac PSM, although it does not need to be. However, there may be few clinically relevant COUs where a PSM would be predictive using a generic heart shape. Computational meshes of the atria, ventricles or whole heart are generated from either MR [13,22] or CT [27,28] images through image segmentation and mesh generation. A typical workflow is to use a contrast-guided region-growing or atlas-registration method to segment the heart surfaces, and then generate an unstructured tetrahedral mesh. For more details see [29]. For applications in which it is important to account for the impact of **scar and BZ**, these are often personalized. Intensity of gadolinium-enhanced MR images is used as input. A common approach is for an analyst to manually identify a region of myocardium, from which voxel intensity mean and standard deviation are computed, and the other voxels are then assigned scar or BZ if voxel intensity is above 3 (scar) or 2 (BZ) times the standard deviation above the mean, although other thresholds or other methods are also used [30].

Accounting for anisotropic propagation in tissue is often necessary in cardiac PSMs, in which case **fiber directions** need to be specified. Typically, a rule-based method is used to generate a fiber map for the patient’s anatomy [13,22,31]. An alternative method is to generate fibers for a different heart using DT-MRI and map those fibers to the patient’s anatomy [19,32]. These approaches could be considered as ‘semi-personalization’; while the fibers fields are not derived directly from patient data, they differ across patients and are dependent on the patient’s specific imaging data. A range of approaches have been used for specifying **tissue conductivities**. Many PSMs do not personalize conductivity and use population values taken from the literature, or fixed conductivities derived by tuning conductivity to match population conduction velocity [16]. Others personalize conductivities using, e.g., non-invasive ECG recordings [33–35] or invasively-gathered intra-cardiac activation maps [36].

Another important choice is the **cell model**, typically either an established human cell model [37–40], or a phenomenological model such as [41,42]. Cell model parameters are typically not personalized (e.g., [13,22]), although in some cases a subset of parameters may be personalized. For example, [34] personalized a time constant in their cell model using the QT interval from ECG recordings. [35] personalized multiple cell model, conductivity and other parameters using ECG recordings. For a phenomenological cell model with a limited total number of parameters, it can be feasible to personalize most or all cell model parameters, for example, recent work in atrial models where all tissue and cell model parameters were simultaneous calibrated for each patient using mapping-system derived local activation times [43,44], or [45] which personalized 21 of 24 cell model parameters in a four-variable cell model.

For PSM-CTs, another factor that varies between implementations is the overall **workflow infrastructure,** including who will be running the simulations for a new patient. There are various possibilities, including (note: in the following, citations are for non-cardiac-EP PSMs): (i) a tool is run by the healthcare provider on local computing resources [46]; (ii) patient data is sent to a remote computing resource (e.g., manufacturer/cloud) and the patient-specific model is automatically generated and run, with results sent back to the healthcare provider [1]; and (iii) patient data is sent to the tool provider who generates and runs the model with an operator performing manual steps.

### 2.2 Verification, validation and uncertainty quantification for cardiac EP PSM

We now consider how VVUQ applies to cardiac EP PSMs. We discuss each of these activities below, in the context of both PSM-CTs and PSM-VCs. To help make the discussion concrete, specific credibility-related questions that arise for cardiac PSMs are listed in Table 1 (PSM-CTs) and 2 (PSM-VCs).

**Table 1:** Example specific credibility-related considerations for patient-specific models as clinical tools. Acronyms: UQ–uncertainty quantification; QOI–quantity of interest; COU–context of use; BZ–border zone.

#	Credibility assessment activity	PSM feature	Specific PSM-CT consideration
1	Verification		What is (expected) numerical error for a new patient in the intended patient population given the chosen mesh resolution?
2	Verification		If there is a remote operator who will do some manual tasks (e.g., in image segmentation or running simulations), what are the potential errors due to intra- or inter-operator variability?
3	Validation		If validation involved a clinical study with an intermediate QOI (see text)–what is the relationship between the intermediate QOI and COU QOI?
4	Validation		If ‘personalized model validation’, that is, validation for each new patient—how relevant are the validation QOIs to the COU QOIs?
5	UQ–model form	Tissue model	Is model form appropriate for all new patients in the intended patient population? Are there sub-populations the tissue model may not adequately represent?
6	UQ–model form	Cell model	Is the cell model appropriate for all patients in intended patient population? Are there sub-populations the cell model may not adequately represent?
7	UQ–personalized parameters	Heart shape	What is the potential error in the personalized heart shape for a new patient and what is the resultant impact on the tool outputs?
8	UQ–personalized parameters	BZ & scar	What is the potential error in the personalized BZ/scar for a new patient and what is the resultant impact on the tool outputs?
10	UQ–personalized parameters	Fibers	If a rule-based method was used—what is the potential error in specified fibers for a new patient and what is the impact on tool outputs?
11	UQ–personalized parameters	Cell model	For personalized cell model parameters—what is the potential error in the personalized values of these parameters for a new patient and what is the resultant impact on the tool outputs?
12	UQ–non- personalized parameters	Conductivity	If a fixed (not personalized) conductivity is used—are the tool outputs insensitive to population variability or other uncertainties in conductivity?
13	UQ–non-personalized parameters	Cell model	For fixed (not personalized) cell model parameters—are the tool outputs insensitive to population variability or other uncertainties in these parameters?

#### 2.2.1 Verification of PSMs

The theory and methods for code verification appear equally applicable to cardiac PSMs as to generic cardiac models. For example, software quality assurance processes (the first ASME credibility factor) are equally applicable to software developed for PSM-CTs and PSM-VCs as for generic models. Similarly, methods developed for demonstrating the monodomain and bidomain equations have been implemented correctly [11,47] apply directly to PSM software.

Calculation verification, on the other hand, introduces some important differences. For example, one option for PSMs that is not applicable to generic models, is the choice on whether to perform a mesh resolution study for each patient or not. With cardiac PSMs, often no verification is performed or a single mesh resolution study is performed to justify the mesh resolution used for all patients. However, in principle it could be performed for all patients. For PSM-VC, this would involve a mesh resolution study for each patient in the cohort (see Table 2, entry 1). For an automated PSM-CT, this could entail the clinical tool automatically performing a mesh resolution analysis to determine the appropriate mesh resolution for each patient. Different possibilities for a PSM-CT could be summarized as: ‘single-patient mesh resolution study’ vs ‘multi-patient mesh resolution study’ vs ‘every-patient automated mesh resolution analysis’. We are not aware of any tool that does the last of these. See Table 1, entry 1.

**Table 2:** Example specific credibility-related considerations for simulation studies that use a virtual cohort of patient-specific models. Acronyms: UQ–uncertainty quantification; QOI–quantity of interest; COU–context of use; BZ–border zone.

#	Credibility assessment activity	PSM feature	Specific PSM-VC consideration
1	Verification		Is discretization error sufficiently small across *all* patients in cohort?
2	Validation		If validation involves only a subset of the virtual patients, how representative are validation subjects to the rest of cohort?
3	Validation		If the validation QOI differs from the COU QOI, how relevant is the validation QOI to the COU QOI?
4	UQ–personalized parameters	Heart shape	Are the study conclusions insensitive to the potential errors in heart shape specification in the virtual cohort?
5	UQ–personalized parameters	BZ and scar	Are the study conclusions insensitive to the potential errors in BZ/scar in the virtual cohort?
6	UQ–personalized parameters	Fibers	Are the study conclusions insensitive to potential error in fiber specification between rule-based fibers and true patient fibers?
7	UQ–personalized parameters	Cell model	For personalized cell model parameters—are the study conclusions insensitive to uncertainty in these parameters?
8	UQ–non-personalized parameters	Conductivity	If a fixed (not personalized) conductivity is used–are the study conclusions insensitive to population variability or other uncertainties in conductivity?
9	UQ–non-personalized parameters	Cell model	For fixed (not personalized) cell model parameters–are the study conclusions insensitive to population variability or other uncertainties in these parameters?

Another part of calculation verification is confirming that use error is minimized. For PSM-VCs, use error considerations are the same as for generic models. However, PSM-CTs can involve manual stages in the overall workflow, for example, in semi-automated image segmentation that must be performed every time a new patient is processed thus resulting in greater potential for use error. Therefore, rigorous verification should involve evaluating potential use error in manual stages when PSMs are generated for new patients. See Table 1, entry 2. Moreover, analogous to clinical image analysis for which intra- and inter-observer variability is routinely evaluated, for PSM-CT workflows with manual stages it may be important to quantify intra- and inter-user variability as part of the verification process.

#### 2.2.2 Validation of PSMs

There is a myriad of possibilities for performing validation of cardiac EP PSMs. Validation may be performed for components within the overall model (e.g., cell model, tissue model, fiber generation method, etc.) or the system-as-a-whole; see [12] for a discussion. Validation could involve comparison against bench, animal, or clinical data (either retrospective or prospective). The validation strategy will depend on the specific COU, the feasibility of validation studies, and the stage of the tool development (e.g., a clinical trial to validate model predictions for a well-developed tool nearing marketing application, versus validation of sub-models against animal data in early-stage development).

Here, we focus on clinical validation of a sufficiently mature PSM. There are many options for clinical validation of PSM. For PSM-CTs, two possible general validation approaches are:

1. A clinical study directly validating the final model-derived tool output, that is, evaluating overall tool performance. For example, a study comparing model-based ablation targets against clinician-chosen ablation targets [13] or a study evaluating the performance of a PSM arrhythmia risk stratification tool that compares the model risk score against a clinical study endpoint of appropriate ICD firing or cardiac death [16]. Such a study could be retrospective or prospective. When prospective, this approach would generally be considered the gold standard model validation for a PSM-CT.
2. A clinical study validating predictions of ‘intermediate’ model outputs that are not the final tool output. For example, a clinical study to validate model predictions of activation patterns, for a tool that computes optimal ablation targets [48], or validation of activation patterns for a tool for guiding CRT lead placement [17]. See Table 1, entry 3.

There is another distinct validation approach that is theoretically possible for PSM-CTs.

3. Performing model validation for *every new patient*. That is, every time the tool is used in the field on a new patient, some patient data is used for personalization (as normal) and some further data is used in validation. This approach, which could be called ‘personalized model validation’, could provide an extra level of confidence that the PSM is able to accurately simulate the specific patient at hand. See Table 1, entry 4. Analysis of the validation results could either be fully automatic, i.e., implemented within the tool software, or overseen by an operator. We are not aware of any PSM tool which does automated personalized model validation. For operator-overseen validation, [44] describe a method for both personalizing and validating patient-specific atrial models using electro-anatomical mapping data, that could be used in a personalize-validate-predict workflow for PSMs for ablation guidance.

For PSM-VCs, some possible validation approaches are:

4. Validate the COU quantity of interest (QOI) for a subset of patients in the virtual cohort: collect clinical measurements of the COU QOI for a subset of the patients in the cohort, and then compare model predictions with clinical measurements for that subset of patients. Then, for the full study, compute the COU QOI for the rest of the virtual patients. For example, in [49] personalized models were developed for seven transcatheter mitral wave replacement patients. Different virtual valve configurations were simulated for each patient. Pre- and post-operative data was available for one patient that served as a validation case. See Table 2, entry 2.
5. Validate a different QOI for all (or some) patients in the virtual cohort: collect clinical measurements of a QOI for all (or some) of the patients in the cohort, and compare with model predictions of that QOI. Then, for the full study, compute the COU QOI. For example, [48] develop a virtual cohort of patient-specific atrial models and validate activation times in each patient, then for the COU simulate atrial fibrillation. This option may be more feasible than the first option. See Table 2, entry 3.
6. Perform cohort/population-level validation: for some QOI, compare distributions or statistics (e.g., mean and standard deviation) predicted across the virtual cohort with the corresponding information for the patient population. This option does not require any patient-level comparison. This could be an alternative, weaker, form of validation if the two options above are not possible, or a complementary addition level of validation.

#### 2.2.3 Uncertainty quantification for PSMs–model form uncertainty

One source of uncertainty in computational models is model form uncertainty, also referred to as model discrepancy. In general, PSMs do not raise unique considerations regarding model form uncertainty. One potential issue arises for PSM-CTs. Since these tools involve applying the modeling workflow to a new patient for which no data is available at the time of model evaluation, it is important to consider whether the chosen model form (tissue model and cell model) will be appropriate for all potential new patients. For example, it is important to consider if there are possible patients in the intended patient population for whom the underlying assumptions (and therefore governing equations) are not appropriate. Reasons for this could be age, sex, or physiological conditions. See Table 1, entries 5–6. However, it is expected that for many applications of cardiac models this will not be a major concern, because the tissue and cell models are typically based on biophysical principles that apply across all sub-populations.

#### 2.2.4 Uncertainty quantification for PSMs–model input uncertainty

**Fig 2:**
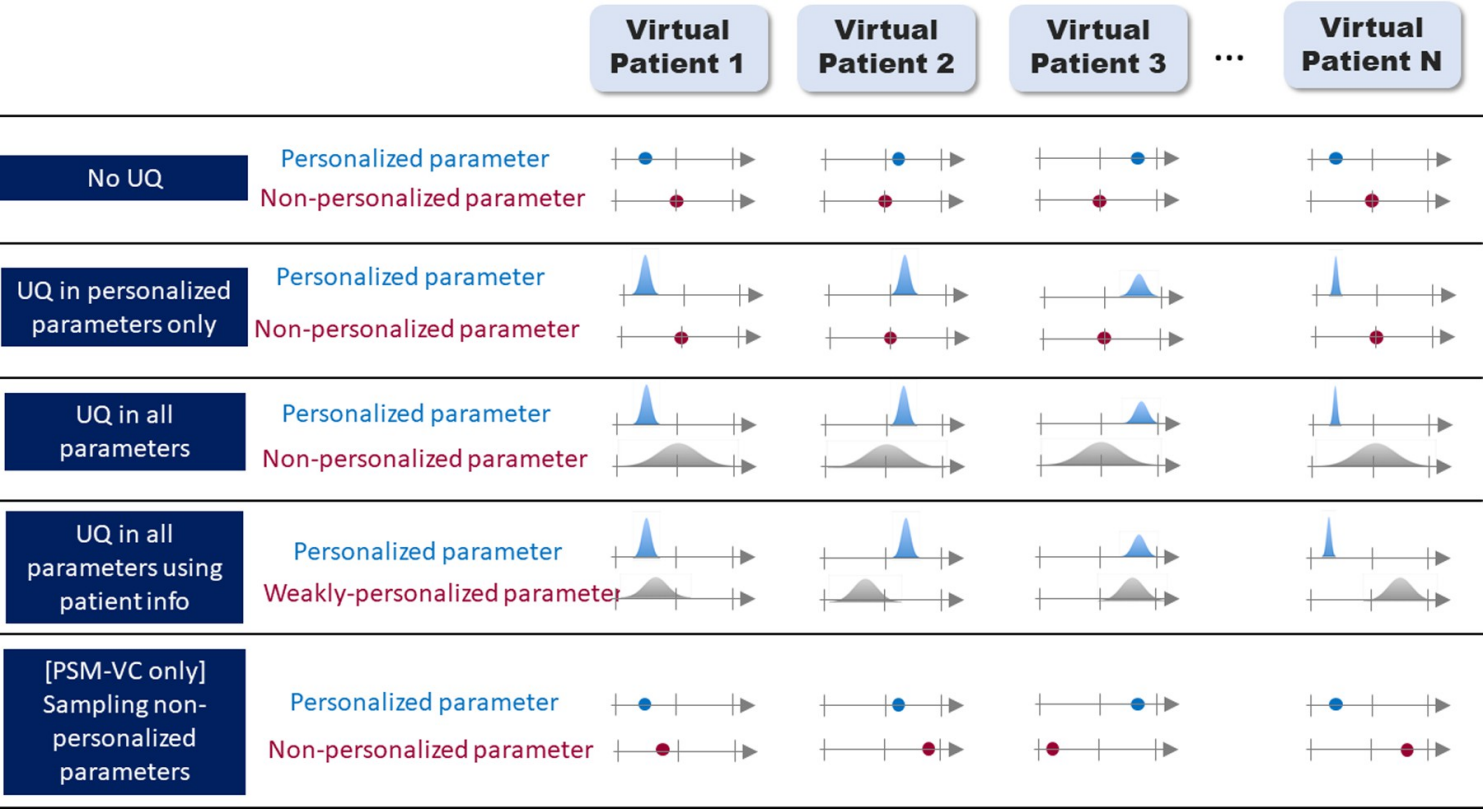
Five example UQ paradigms in patient-specific modeling. The N virtual patients could be N members of a virtual cohort in PSM-VC, or N new patients for a PSM-CT. In the first row, there is no UQ; the personalized parameter varies across the subjects, the non-personalized parameter is constant. In the second row, uncertainty in the personalized parameter (e.g., due to measurement error) is accounted for–represented here as a probability distribution, though other methods for accounting for uncertainty could be used. In the third row, uncertainty in the non-personalized parameter (e.g., due to population variability) is also accounted for. In the fourth row, some patient information is used to constrain the distributions for the ‘non-personalized’ parameters (e.g., if the parameter is known to differ between sexes). In the final row (PSM-VC only) the non-personalized parameter is randomly sampled in the cohort members, rather than taking the population average value for all patients. Other options are possible.

Finally, we consider what it means to perform UQ on model inputs for PSMs. Model inputs can be either personalized or fixed; we consider each in turn for both PSM-CT and PSM-VC. To aid the discussion, Fig 2 illustrates different UQ paradigms that are possible with PSMs.

For PSM-CT (see Fig 2):

- For personalized inputs: For each new patient, there will be some potential error between the obtained personalized value and the patient’s true value for that input, due to instrument measurement error, image acquisition or segmentation errors, calibration uncertainty, etc. For example, the patient’s heart shape will differ slightly from the personalized geometrical mesh. UQ in personalized inputs involves characterizing this uncertainty and evaluating its impact on tool outputs (illustrated in Fig 2 row 2). It is important that a PSM-CT does not make different clinical recommendations (e.g., implant vs do not implant a defibrillator) for different input values within the patient’s uncertainty for that input. For example, if a personalized parameter in a PSM-CT was measured to be *a*±δ for a patient, with δ sufficiently large such that the tool provides different recommendation using *a*+δ vs *a*-δ, the tool would not be reliable for that patient. See Table 1, entries 7–11.
- Non-personalized inputs: For non-personalized parameters, a fixed value is typically used for every patient. However, the true (unknown) value of the parameter for a patient could be any value within the range of values observed in the patient population. If sensitivity analysis was performed as part of tool development and it was determined that this parameter did not impact the COU QOI and therefore could be set to a fixed value rather than personalized, then no UQ needs to be performed. If not, it may be important to consider how much the uncertainty (e.g., due to population variability; illustrated in Fig 2 row 3) in this parameter could impact the COU QOI. See Table 1, entries 12–13. However, a tool can show good results in performance testing with non-personalized parameters fixed to a single value in all patients.

Typically, when a PSM-CT is applied to a new patient, no UQ is performed (Fig 2 row 1) due to the computational expense of UQ. However, UQ may be performed in the tool development stage.

For PSM-VCs (see Fig 2):

- For personalized inputs: Any errors in personalized inputs in PSM-VCs are arguably less important than for PSM-CTs, because while the resultant virtual patient may not match the *real* patient exactly, it may still be a good representative sample from the patient population and therefore suitable for the virtual cohort. However, it is critically important that overall study conclusions are not sensitive to these uncertainties. See Table 2, entries 4–7.
- For non-personalized inputs: For PSM-VCs, as with PSM-CTs, if the parameter was held fixed because previous sensitivity analysis had shown the model was insensitive to this parameter, across values found in the population, then no further UQ need be considered. However, if the COU QOI is sensitive to the parameter, a virtual cohort with all patients taking the same value for this parameter may not be representative of the full patient population. One option here is to do full UQ, either using a probability distribution representing population variability (Fig 2 row 3) or a probability distribution representing population variability conditional on known information about patient (e.g., on sex) (Fig 2 row 4). These options are very computationally expensive. An intermediate option is to assign one randomly sampled value to each patient. In this case each virtual patient may not match the true patients but the virtual cohort may be more representative of true population (Fig 2 row 5). (Note: we do not necessarily recommend this option, since for each patient it samples from a distribution with *n = 1*, it is mentioned only to illustrate how there are a myriad of possible approaches for PSMs). Whichever approach is taken, we again suggest that the aim of the UQ be to demonstrate that the overall study conclusions are not sensitive to the input uncertainty. See Table 2, entries 8–9.

## 3. Simulation studies

In this section we present two simulation studies related to verification and uncertainty quantification using a virtual cohort of 24 left ventricular PSMs. The results of these studies will inform the verification and uncertainty quantification sections of Section 4.

### 3.1 Multi-patient mesh convergence study

In Section 2 we identified multi-patient or ‘every-patient’ mesh resolution studies as analyses that are not typically performed in cardiac PSMs. The aim of this first study is to quantify how much errors related to spatial discretization can vary across patients. This will provide information about the importance of performing multi-patient/every-patient mesh resolution studies in PSM-CTs or PSM-VCs (Table 1 entry 1 and Table 2 entry 1). Using the virtual cohort, we computed discretization error in predicted global activation times and simulated ECGs resulting from apical pacing, using meshes of different resolutions for each patient, and determined if discretization error varied across patients.

**Fig 3:**
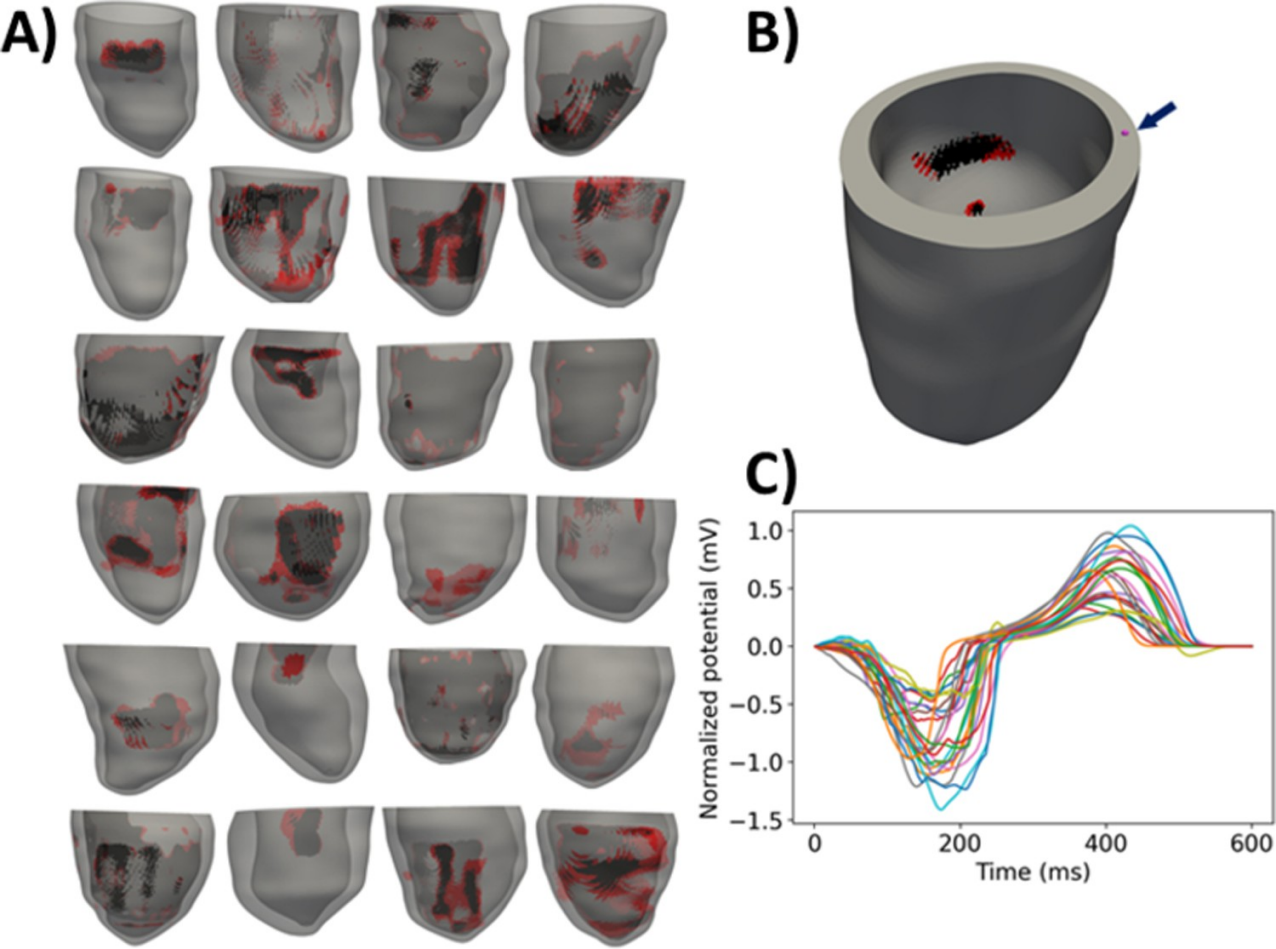
A): left ventricular meshes for 24 patients (red–border zone; black–scar). B) base timing location for one patient. C) simulated V1 ECG for all patients (note: apical pacing not sinus rhythm).

Full methods details are provided in S1 Text (Section S1). Briefly, we used 24 publicly-available patient-specific ventricular models, each with personalized anatomy, and regions of scar and BZ [50] (see Fig 3). Details on the development of these meshes are provided in [22]. Each model has fibers generated using a rule-based method [51]. We simulated electrical activity in the ventricles by solving the monodomain equations with the ten Tusscher et al. cell model [37], using the cardiac EP simulator Chaste [52]. For each patient, four meshes of increasing resolution were generated, referred to as: very low, low, medium and high resolution. Average edge lengths and number of elements for each group of meshes are provided in Table 3. Note that the average edge length for the meshes named ‘high-resolution’ is 275 microns, greater than the 100 micron range often considered as a high resolution in the field [47].

**Table 3:** Details on meshes used in mesh resolution study.

	Average edge length (mean ± SD across patients, microns)	Number of elements (mean ± SD across patients, millions of elements)
Very low	1029 ± 1.6	1.15 ± 0.28
Low	630 ± 0.1	5.57 ±1.35
Medium	426 ± 0.5	16.7 ± 4.1
High	275 ± 0.02	69.1 ± 16.9

Each mesh for each patient was paced for one beat at the apical location, and two quantities were measured. First, for each patient a location on the anterior portion of the base, midway across the tissue, was selected and the activation time of that node was computed, scaled by distance between that node and the apical pacing node to account for different heart sizes. Second, for each patient, the electrode locations for the 12 lead ECG were approximated by aligning the hearts (by the long- and short-axes, based on Universal Ventricular Coordinate system) with a heart within an existing full torso computational model [53] for which 12-lead ECG electrode locations were already defined, as in [20]. The ECG at the V1 lead was computed using the method of [54]. Errors in normalized activation time and ECG were computed for the very low, low and medium resolution meshes using the high-resolution results as ground truth. ECG error was quantified using a scaled infinity (max) norm:

$$E_{x}={\left\|\phi_{X}-\phi_{H}\right\|}_{\infty }/{\left\|\phi_{H}\right\|}_{\infty }$$

where *ϕ_X_* is potential for the mesh of interest (very low/low/medium) and *ϕ_H_* the result of the high-resolution mesh. Since the computed ECGs on different resolution meshes were similar in shape but exhibited a temporal lag, we also estimated this lag by finding the shift Δt which maximized the cross-correlation between the two ECGs. Simulations were computationally expensive, approximately 2 core-years, due to the high resolution meshes and number of patients.

**Fig 4:**
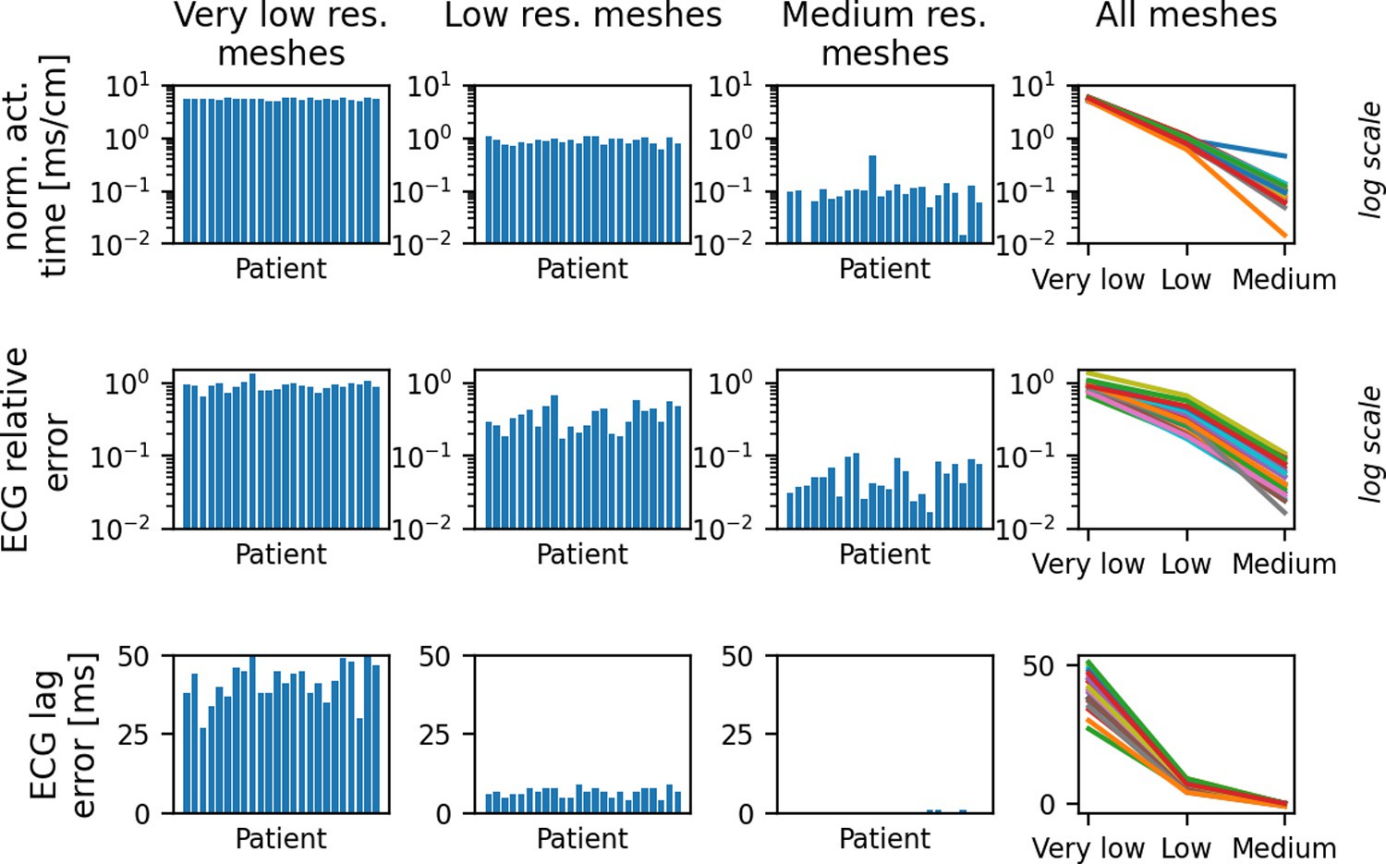
Discretization errors (defined as the difference between very low/low/medium results and the high-resolution results), for each patient, in normalized apex-to-base activation time (top row) and simulated ECG (middle and bottom rows). Note: Log scale used for top and middle rows. ECG lag errors (bottom row) take integer values, and since most results are exactly zero for the medium resolution meshes, a log scale is not used for ECG lag.

Fig 4 and Table 4 presents the results of this study. For the very low and low resolution meshes, while there is some variability in discretization error across the patients, we did not observe large coefficients of variation for the very low or low resolution meshes (Table 4). Very large coefficients of variation on the lower resolution meshes would have indicated that multi-patient mesh resolution studies could be critically important in minimizing patient risk for PSM-CTs, because a single-patient mesh resolution study might erroneously conclude the low/very low resolutions are acceptable, which may not be true for new patients. However, more variability was observed on the medium resolution meshes. For example, coefficients of variation are 0.87 for normalized activation time and 0.48 for relative ECG error. For activation time and the medium resolution meshes, there was also an outlier patient with a significantly greater error than the other patients (patient 11, error = 0.46 ms/cm; all others < 0.14 ms/cm). The ECG relative errors on the medium resolution meshes vary across patients between 1.7% to 10.9%. These results demonstrate how a single-patient mesh resolution study could lead to an erroneous conclusion regarding appropriate step-sizes for other patients. It is not clear why the outlier case presented significantly higher error than the other patients and further investigation is needed to identify patient/mesh characteristics that could lead to heightened error. We note also that if a precise estimate of discretization error is required, for example as part of a comprehensive uncertainty analysis that accounts for all sources of uncertainty, discretization errors may need to be estimated for each patient, especially on higher resolution meshes.

**Table 4:** Discretization error coefficients of variation (COV), across patients, using different resolution meshes (columns) for normalized apex-to-base activation time and simulated ECG.

	Very low resolution meshes	Low resolution meshes	Medium resolution meshes
COV for error in normalized activation time	0.05	0.14	0.78
COV for ECG relative error	0.15	0.38	0.48
COV for ECG lag error	0.14	0.21	2.64

### 3.2 Impact of uncertainty in personalized border zone regions in a virtual cohort simulation study

In Section 2.2 we suggested that UQ for PSM-VCs should be based around demonstrating that the conclusion of the study is insensitive to underlying uncertainties. To investigate how this would work in practice, we investigated if the conclusions of a recently published study [22] are insensitive to uncertainty in key model inputs.

**Fig 5:**
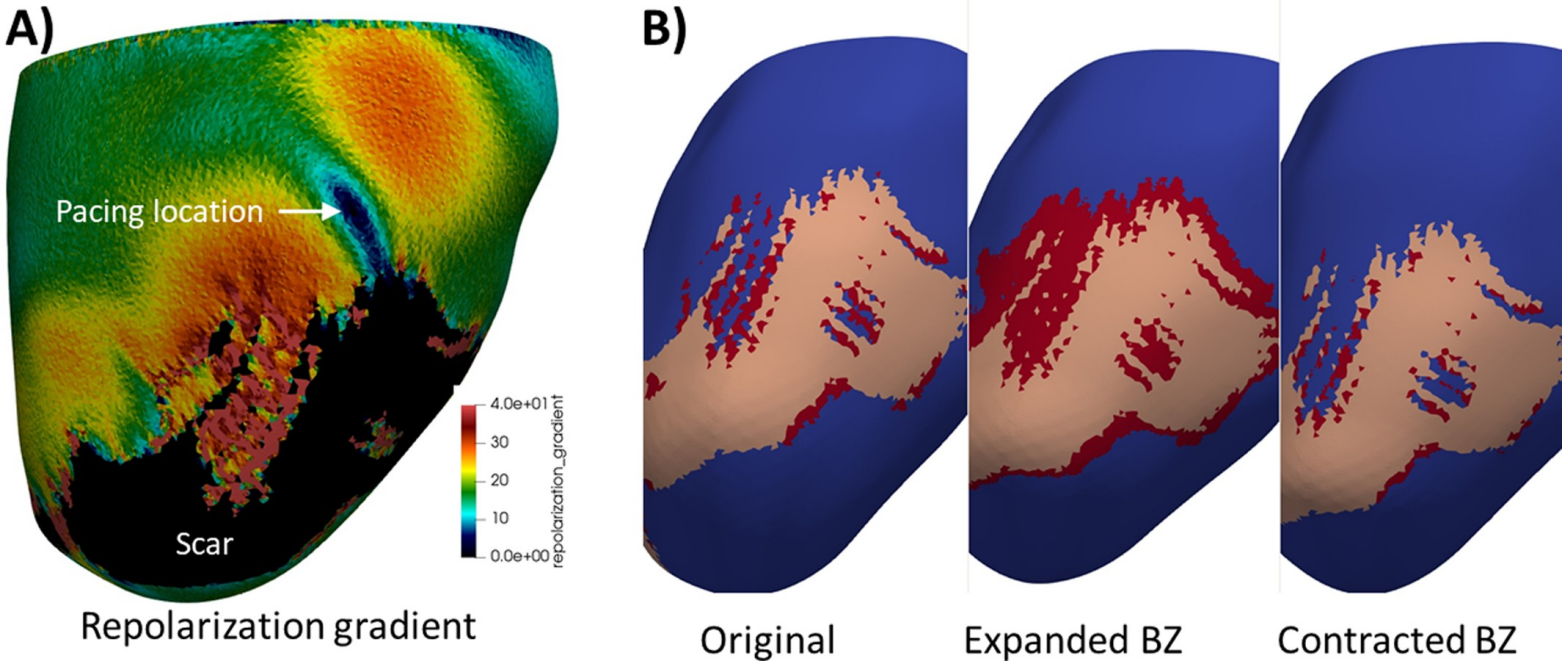
A) repolarization gradient map for sample patient, pacing 0.2cm from scar. Colors represent repolarization gradient except scar is colored black. B) original mesh and meshes with expanded and contracted border zone for the same patient. Tissue is blue, BZ is red, scar is pink.

**Fig 6:**
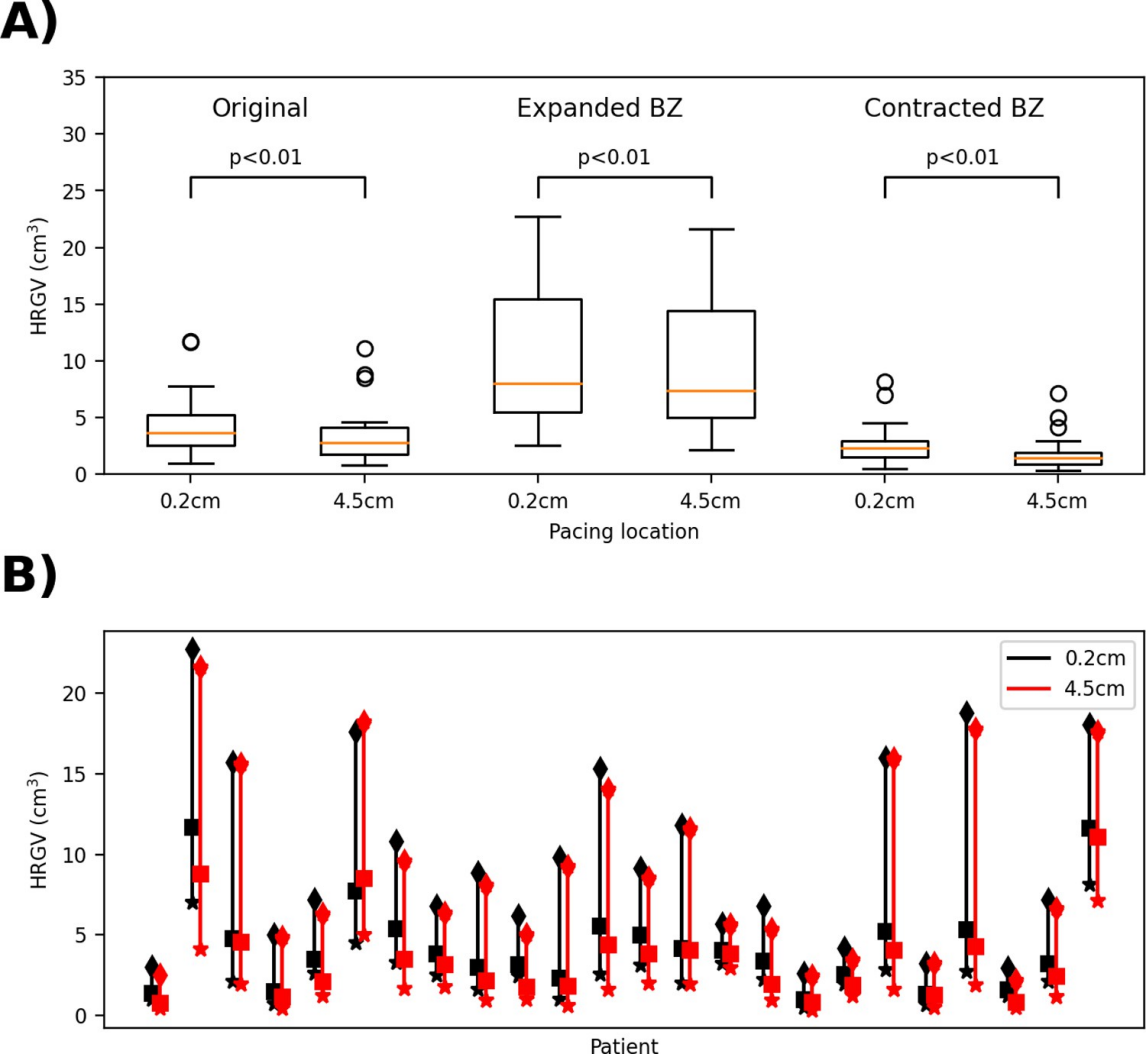
Impact of uncertainty in border zone extent on high repolarization gradient volumes (HRGV). A): boxplots comparing pacing at 0.2cm from scar with pacing 4.5cm from scar for each case. B): Range of HRGV values across each of the three cases (contracted BZ (stars), baseline (squares), expanded BZ (diamonds)) for each patient.

This previous study demonstrated that high repolarization gradients in the vicinity of scar, which is a surrogate for CRT-induced ventricular tachycardia risk, are more likely when the tissue is paced in proximity to scar vs distant to it. Following [22], we define high repolarization gradient volume (HRGV) as the volume of tissue or BZ within 1cm of scar for which repolarization gradient magnitude was greater than a threshold value of 30ms/cm. In [22], boxplots showed a trend of decreasing HRGV pacing 4.5cm from scar compared to 0.2 cm, confirmed using a one-sided paired t-test. In an attempt to reproduce this conclusion, we used the medium resolution meshes above, and simulated one beat of activity after pacing at locations 0.2 cm and 4.5cm from scar (specific locations same as used in [22]). For each node in the mesh, the repolarization time (time for membrane voltage to return to below -70 mV) was computed, and repolarization gradients were calculated as the magnitude of the spatial gradient of repolarization time. Fig 5A plots the repolarization gradient for a sample patient for the 0.2cm case. We computed HRGV for each patient paced either 0.2cm or 4.5cm from scar. A one-sided paired t-test was performed to confirm the hypothesis that HRGV is greater pacing at 0.2cm compared to 4.5cm (p<0.01) (see Fig 6A, below, ‘Original’).

We aimed to study impact of uncertainty in one personalized and one non-personalized input. To demonstrate this concept, we selected two parameters that were expected to influence the study outcomes, although alternately a full variational-based global sensitivity analysis could have been used to systematically identify important parameters. For personalized input, we selected border zone size, since HRGV is defined using the region within 1cm of scar (i.e., regions of significant BZ) and changes in conductivity from tissue to BZ are expected to impact HRGV. For the non-personalized input, we selected tissue conductivity since depolarization and repolarization gradients are directly impacted by conductivity. First, to determine if the study conclusion is robust to measurement uncertainty in the BZ specification for each patient, we ran simulations with altered BZ. The uncertainty in BZ–the difference between prescribed BZ and true unknown BZ–is due to potential errors in both the image acquisition and image segmentation stages and difficult to quantify, with very little information available in the literature. BZ was originally identified as regions with signal intensity 2–3 standard deviations above mean signal intensity within healthy myocardium. Other methods are possible, including different standard deviations (generally larger SDs, corresponding to less BZ) or the full-width half-maximum (FWHM) method [55]. One approach to investigating sensitivity to BZ uncertainty would be to re-segment the images using different segmentation methods and perform simulations on new meshes. However, we did not have access to the raw imaging data and in any case such an approach could only account for uncertainty due to image segmentation uncertainties, not image acquisition. Instead, we chose to focus on potential errors in BZ extent, neglecting potential errors in BZ morphology, and varied the BZ extent by growing or shrinking BZ relative to surrounding myocardium. Scar region was not altered since the pacing locations are based on distance to scar.

First, we computed statistics on the thickness of the BZ layer across the patients. Average BZ thickness in each heart was 1.2 ± 1.3 mm (mean ± SD across patients), maximum thickness was 8.9 ± 6.2 mm. The following perturbations were then made for each patient: BZ region contracted by 0.5mm (all BZ elements within 0.5 mm of myocardium converted to myocardium elements), and BZ expanded by 1mm (all tissue elements within 1mm of BZ or scar converted to BZ). See Fig 5B. These choices of 0.5mm and 1mm are motivated by: (i) the computed average BZ width across patients; (ii) the voxel size in the original images (0.6–1.4 mm in-plane, 8-20mm out-of-plane), and (iii) the resultant BZ volumes for patient 1, which was 0.29 cm^3^ (contracted BZ), 0.73 cm^3^ (original BZ), 2.33 cm^3^ (expanded BZ). This relative range of volumes is comparable to the relative range of BZ volumes using different imaging methods provided in [55]. Overall, we believe the contracted and expanded BZ cover a reasonable range of plausible errors in personalized BZ extent.

For each of the two new meshes for each patient, HRGV was re-calculated pacing from the same sites as previously. Fig 6A provides boxplots for the original, expanded BZ and contracted BZ cases. We consider the following cases:

1. Assuming *systematic* error in BZ specification, with BZ in all patients over-estimated: to assess if the study conclusions are robust to these errors, we repeated the t-test using the contracted BZ results only, differences remained statistically significant (p<0.01).
2. Assuming *systematic* error in BZ specification, with BZ in all patients under-estimated: for this case we repeated the t-test using the expanded BZ results only, differences remained statistically significant (p<0.01).
3. Assuming *unbiased* error in BZ specification across patients: here, the 3 sets of results for each patient (original, contracted, expanded BZ) represent three samples that we assume are equally likely for each patient, and uncorrelated across patients; 3^24^ possible combinations in total. We initially performed a t-test using the maximum patient HRGV value from the 0.2cm results and the minimum patient HRGV value from the 4.5cm results. This is not a set of results that could ever be attained, since it uses different patient BZ representations for the different pacing sites, but if statistically significant, would be sufficient to guarantee that all 3^24^ possible cases are statistically significant. However, the result of this t-test was not statistically significant. Therefore, we randomly sampled N = 10^6^ sets of results from the 3^24^ possibilities. Every case was statistically significant (p<0.01).

Overall, we conclude that the study conclusion is robust to measurement error in BZ extent. In contrast, the absolute value of HRGV across patients is heavily impacted by BZ uncertainty (Fig 6B). Therefore, this is an example where the absolute value of the QOI, HRGV, is heavily impacted by uncertainty in a personalized input, and would not be a reliable quantity on which to base patient-specific decisions (in a hypothetical PSM-CT), but the QOI trends analyzed using a virtual cohort are insensitive to the input uncertainties.

**Fig 7:**
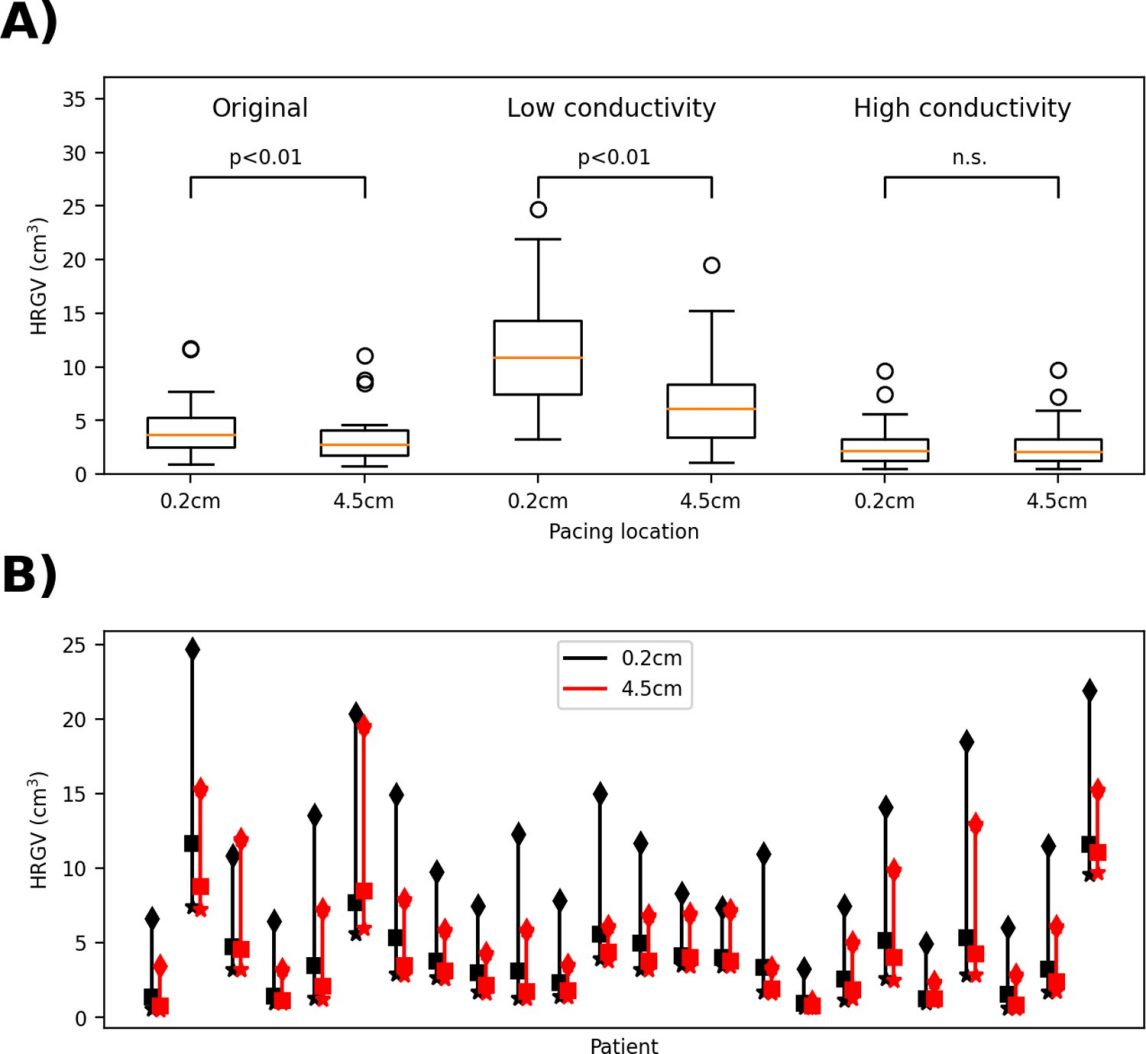
Impact of uncertainty in tissue conductivity on high repolarization gradient volumes (HRGV). A): boxplots comparing pacing at 0.2cm from scar with pacing 4.5cm from scar for each case. B): Range of HRGV values across each of the three cases (low (diamonds), baseline (squares), high conductivity (stars)) for each patient.

Conductivity, unlike BZ, is a non-personalized input and took a constant value across all patients. There are numerous factors which impact baseline tissue conductivity, including population variability, tissue heterogeneity, and impact of heart disease. To generate conductivities covering a reasonable range of values, we computed fiber conductivities corresponding to conduction velocities that were 80% and 120% of the baseline conduction velocity (see S1 Text, Section S1 for details). We repeated the simulation study with these low and high conductivities, retaining the same anisotropy ratio between fiber and sheet conductivities. Results are provided in Fig 7. Following our approach for BZ:

1. Assuming systematic error in conductivity, all patients over-estimated: Using the low conductivity results, differences remained statistically significant (p<0.01).
2. Assuming systematic error in conductivity, all patients under-estimated: Using the high conductivity results, differences were not statistically significant. However, this is likely due to higher conductivity leading to lower repolarization gradients everywhere in the heart; with a lower choice of threshold value of 25ms/cm there is a statistically significant difference.
3. Assuming unbiased error in conductivity across patients: using the same approach as for BZ, randomly sampling N = 10^6^ possibilities, 96.5% of cases were statistically significant. This is a conservative estimate however, since it assumes all three conduction velocities (low, baseline, high) are equally likely for each patient. Using a more realistic representation of our uncertainty in patient conduction velocity centered on the baseline CV of 66cm/ms, *CV* ~ *N*(66, 6.6^2^) (so that the low and high values correspond to a 95% confidence interval), we randomly sampled independent CVs for each patient, linearly interpolated HRGV, and generated N = 10^6^ sets of results. Here, 99.995% of cases were statistically significant.

We conclude that the findings of [22] are likely robust to uncertainty in tissue conductivity. Overall, while there are various limitations to this study (subjectivity in choice of key inputs, lack of analysis of impact of BZ morphology uncertainty, somewhat arbitrary choice of limits for conduction velocity), these results support the conclusions of [22] and illustrate how UQ focused on virtual cohort study conclusions can be performed.

## 4. Evaluating patient-specific models using ASME V&V40

In this section we use the results of Sections 2 and 3 to identify how important characteristics of PSMs can be considered when assessing credibility with the approach of ASME V&V40. We consider how PSM characteristics apply to ASME V&V40 credibility factors and provide example gradations for PSMs (recall that ASME V&V40 requires the user to define gradations; all gradations we provide are examples only). Given the differences between PSM-CTs vs PSM-VCs as discussed in Section 2, we consider both cases separately. The below is not cardiac EP specific and we expect it to be applicable to PSMs in many other disciplines, especially other medical-imaging based PSMs. However, since we have only considered cardiac PSMs there may be missing considerations for other disciplines. Table 5 summarizes our observations which are discussed below.

**Table 5:** Summary of observations on considerations for PSM credibility assessment in relation to V&V40 credibility factors and gradations. PSMs as clinical tools (PSM-CT) or PSM virtual cohort studies (PSM-VC) are considered separately.

Category	V&V40 Credibility factor	PSM-CT	PSM-VC
Verification	Software quality assurance	No unique PSM considerations identified.	
	Numerical code verification		
	Discretization error	Gradations could account for number of patients these activities are performed on (see Table 6 & S1 Text, Section S2).	
	Numerical solver Error		
	Use error	For PSMs, with manual stages, there is a possibility of user variability related to subjectively chosen inputs. See S1 Text, Section S2 for example gradation accounting for this.	No unique PSM considerations identified.
Validation–model	Model form	No unique PSM considerations identified.	
	Model inputs–quantification of sensitivities	For PSMs, sensitivity analysis and uncertainty quantification are intimately linked, so an alternative approach is have a single sensitivity analysis and uncertainty quantification factor with possible subfactors: • Inputs analyzed • Rigor of input uncertainty characterization • Number of patients • Output quantities analyzed	
	Model inputs–quantification of uncertainties		
Validation -comparator	Quantity of Test Samples	There may be unique PSM considerations, but these will be dependent on the specific validation activities performed. For some cases where a PSM is validated against clinical data, these factors could be interpreted as: • Number of validation subjects • Range of characteristics of validation subjects • Patient data • Patient measurements See S1 Text, Section S2 for example gradations.	
	Range of Characteristics of Test Samples		
	Characteristics of Test Samples		
	Measurements of Test Samples		
	Quantity of Test Conditions	There may be unique PSM considerations, but these will be dependent on the specific validation activities performed.	
	Range of Test Conditions		
	Measurements of Test Conditions		
	Uncertainty of Test Condition Measurements		
Validation—comparison	Equivalency of Input Parameters	No unique PSM considerations identified.	
	Output Comparison–quantity		
	Equivalency of Output Parameters		
	Agreement of Output Comparison		
	Rigor of Output Comparison		
Applicability	Relevance of the QOIs		
	Relevance of the Validation Activities to the COU	For PSMs, assessing the relevance of the validation subjects to the full patient population / full virtual cohort is a component of applicability assessment	

For code verification credibility factors, *software quality assurance* and *numerical code verification*, our review in Section 2.2.1 does not raise any unique PSM considerations. However, for calculation verification, we discussed in Section 2.2.1 and demonstrated in Section 3.1 how calculation verification results can vary across patients, which means it may be important to consider number and range of patients when assessing *discretization error* and *numerical solver error*. Example gradations for the *discretization error* and *numerical solver* credibility factor are provided in Table 6 and S1 Text, Section S2, respectively, that account for these.

**Table 6:** Original V&V40 gradation for the ‘Discretization Error’ credibility factor, and example possible gradations for PSM-CT and PSM-VC. Changes highlighted in bold. [Original V&V40 example gradations reprinted from ASME V&V 40–2018, by permission of The American Society of Mechanical Engineers. All rights reserved].

Example gradation in ASME V&V40	Possible gradation for PSM-CT	Possible gradation for PSM-VC
(a) No grid or time-step convergence analysis was performed to estimate the discretization error. (b) Applicable grid or time-step convergence analyses were performed and their respective convergence behaviors were observed to be stable, but the discretization error was not estimated. (c) Applicable grid or time-step convergence analyses were performed and discretization error was estimated.	(a) No grid or time-step convergence analysis was performed to estimate the discretization error. (b) Applicable grid or time-step convergence analyses were performed, **using one or a small number of patients,** and their respective convergence behaviors were observed to be stable, but the discretization error was not estimated. (c) Applicable grid or time-step convergence analyses were performed and discretization error was estimated, **using one or a small number of patients.** **(d) Applicable grid or time-step convergence analyses were performed and discretization error was estimated using a range of representative patients** **(e) Applicable grid or time-step convergence analyses are automatically performed on each new patient, and discretization error is estimated, and used in processing the results.**	(a) No grid or time-step convergence analysis was performed to estimate the discretization error. (b) Applicable grid or time-step convergence analyses were performed and their respective convergence behaviors were observed to be stable, but the discretization error was not estimated, **using one or a small number of subjects.** (c) Applicable grid or time-step convergence analyses were performed and discretization error was estimated, **using one or a small number of subjects.** **(d) Applicable grid or time-step convergence analyses were performed and discretization error was estimated, using a range of patients covering the virtual cohort.**

Regarding the *use error* credibility factor, we discussed in Section 2.2.1 how for PSM-CTs with manual stages, the user (either clinician or remote operator) may need to make subjective decisions for some inputs (e.g., in the image segmentation stage), in which case there is potential for both intra- and inter-user variability. Therefore, for PSM-CT with manual stages, this is a potential source of unreliability. To ensure that this is accounted for, a use error gradation could include assessment of both intra- and inter-user variability at the higher levels of rigor. Alternatively, the factor could be broken down in two sub-factors, for example *use error–objective inputs* and *use error–subjectively-chosen inputs*. An example gradations for the latter is provided in S1 Text, Section S2.

Next, we consider the ‘*validation–model*’ credibility factors. For the *model form* credibility factor our review in Section 2.2.3 does not raise any unique PSM considerations. However, we observed in our review in Section 2.2.3 the myriad possibilities for performing sensitivity analysis (SA) and uncertainty quantification (UQ) for PSMs, and discussed the importance of distinguishing between SA/UQ for personalized vs non-personalized inputs. We performed a virtual cohort UQ study in Section 3.2, which illustrates how the level of rigor in SA/UQ is dependent on the number of inputs analyzed, the rigor in quantifying the input uncertainty, the number of patients considered, and the outputs considered. One option is to define a one gradation which covers all of these; an alternative option is to define a single SA/UQ credibility factor with multiple sub-factors. S1 Text, Section S2 provides example gradations for the latter option.

The next credibility factors are those related to the comparator. ASME V&V40 defines comparator credibility factors related to ‘*Test samples*’ and ‘*Test conditions’*. Each are broken into 4 sub-factors, listed in Table 5. In Section 2.2 we discussed how there are a variety of approaches that can be taken to validate a PSM-CT or a PSM-VC, with three examples provided for each. In situations such as cases 1, 2, 4 and 5 in Section 2.2, the *test samples* sub-factors could be interpreted as listed in Table 5. For example, the ‘*quantity of test samples’* sub-factor corresponds to the number of validation subjects used. Example gradations for these sub-factors are provided in S1 Text, Section S2. However, in general, how to interpret the comparator sub-factors, and appropriate gradations, will be dependent on the specific validation activities chosen. The same applies for the *test conditions* sub-factors. For medical device models, greater credibility is possible when the validation experiments subject the device under test to a wide range of external conditions (e.g., external loading or heating). In clinical studies, the feasibility of controlling test conditions will vary significantly between studies; for many studies it may not be possible or ethical to vary the imposed condition. Consequently, we have not attempted to provide example gradations for the test conditions sub-factors because we expect appropriate gradations to be heavily dependent on the specific validation activities performed.

For the remaining validation credibility factors (*‘validation comparison’*), no unique PSM considerations were identified from Sections 2 and 3.

Finally, considering applicability credibility factors, for the factor “*relevance of validation activities to COU”*, there may be unique PSM-specific considerations, though it depends on the specific validation approach chosen. When validating against clinical data, an applicability question is “*how representative are the validation subjects to the full patient population*?” (PSM-CT; cases 1–3 in Section 2.2) or “*how representative are the validation subjects to the full virtual cohort*?*”* (PSM-VC; cases 4–5 in Section 2.2). A gradation for the *relevance of validation activities to COU factor* could be defined to account for this. Alternatively, a new applicability sub-factor “*Representativeness of validation subjects*” could be defined.

## 5. Discussion

Patient-specific modeling is a new frontier in computational modeling for which the topic of credibility assessment has largely been unexplored. While there are many publications evaluating the predictive capability of a particular patient-specific model [56–59], we are unaware of any previous work on the general topic of PSM credibility. Here we address this gap by providing the first, to our knowledge, general treatise on credibility assessment of general PSMs, within our scope of medical image based PSMs. Capitalizing on the maturity of cardiac modeling, we used this field as an exemplar to understand the nuance and complexities of evaluating PSMs.

First, we reviewed methods utilized in the development of cardiac PSM, and applications of these models. We determined that the differences between PSMs workflows developed as clinical tools (PSM-CTs) vs a set of pre-computed PSMs forming a virtual cohort (PSM-VC), are so fundamental that all the subsequent analysis and discussion should distinguish between these two cases. The two cases are not comprehensive; other applications of PSMs that do not fit neatly into either category are possible. However, all publications reviewed fell into one of these categories, and important PSMs from other fields do so as well, for example Heartflow [1] is a PSM-CT and the Virtual Family [60] is a PSM-VC. Our review illuminated the range of approaches possible for generating PSMs.

We then considered what each of verification, validation and uncertainty quantification means for PSMs. Verification was relatively straightforward, although we identified the importance of assessing error arising from inter- and intra-user variability for manual stages of PSM workflows. It is more complicated to characterize the validation and UQ process for PSMs. There are many potential approaches that could be taken to validate a PSM (some examples are provided in Section 2), and even categorizing these approaches is challenging, let alone defining general rules of good practice. Similarly, there is a range of options for performing UQ for a patient-specific model, and numerous choices need to be made, such as which inputs to explore (anatomical inputs, material parameters, functional parameters; personalized or non-personalized); how to estimate the input uncertainty; how many patients to consider; and what outputs should be analyzed. We emphasized the importance that any PSM-CT-derived clinical recommendation (e.g., implant an ICD vs do not impact an ICD) is insensitive to uncertainty in personalized inputs, and any PSM-VC-derived conclusion is insensitive to input uncertainties.

In Section 3.1 we used a set of 24 personalized ventricular models to investigate how discretization errors vary across patients. Large differences in errors between patients would have indicated that single-patient mesh resolution studies are not sufficient to ensure credibility of PSMs (especially for high-risk applications). While we did not observe large differences in errors between patients on the lower resolution meshes, there was more variability for the higher resolution meshes. Of course, conclusions may be different for different models or different outputs; similar analyses for other cardiac EP outputs of interest, and for other PSMs, need to be performed before general conclusions can be drawn about the importance of multi-patient verification with PSMs. In general, our results support the need for multi-patient verification for higher-risk applications of PSMs, due to the variability observed using the higher-resolution meshes. Still, conducting mesh resolution studies with multiple patients is computationally expensive and for lower-risk applications there is a trade-off between insight gained vs computational expense.

In Section 3.2 we explored the process of demonstrating that scientific results using a PSM-VC are not impacted by input uncertainty. We considered two inputs, one personalized (border zone region) and one non-personalized (conductivity). We first reproduced the findings of [22] that pacing in vicinity to scar increases repolarization dispersion versus distant pacing. (Note that we used the same geometries and pacing sites as [22], but different models, meshes and numerical solvers (see S1 Text, Section S1), and therefore being able to reproduce the findings of [22] demonstrates that with public data sets it is possible to reproduce simulation study results across platforms). In our statistical analysis of the results, we emphasized that performing a statistical test with only a perturbed set of results (e.g., only with the expanded BZ results) implicitly assumes that the BZ measurement error is biased in the same direction for all patients. A perhaps more likely case is unbiased errors–e.g., BZ too large for some patients, too small for others–which requires a statistical test accounting for uncertainty in measurements. We used a sampling approach to achieve this, but other methods are possible, see [61] and references therein for a discussion.

It is interesting to observe that the uncertainty in the non-personalized input (conductivity) led to similar or greater output uncertainty compared to the personalized input (BZ extent). For PSMs, the ideal scenario is to choose which parameters to personalize based on sensitivity analysis during early-stage model development. Parameters that do not impact the output, when varied across their population range, do not need to be personalized, other parameters should be personalized. However, this is typically not feasible, instead which parameters are personalized is at least partially motivated by data-collection constraints. Therefore, post-development UQ, as performed here, may reveal if uncertainty in the fixed (non-personalized) inputs impacts predictions. That was indeed the case in this example. The gradations developed in Section 4 account for this observation.

In Section 4 we identified how important characteristics of PSMs can be considered when assessing credibility with the approach of ASME V&V40. This section was based on our findings for cardiac models in Sections 2 and 3, but we expect it to be relevant to other medical image based PSMs. We considered which ASME credibility factors have unique PSM-related considerations, including providing example gradations for some factors, that cover a range of activities that could be performed when evaluating a PSM. General observations were mostly constrained to the verification and UQ related credibility factors. PSM considerations for validation will be heavily dependent on the specific validation approach taken. Since Section 4 was based on our review of cardiac PSMs in Section 2 and the two case studies in Section 3, there is potential for improving or refining the recommendations of Section 4 based on review of other modeling fields and results from further case studies, cardiac and otherwise. We hope such efforts are pursued in the future so that the relative importance of different credibility activities for PSMs continues to be uncovered. Overall, we believe the results of this paper will be useful to developers of cardiac and other medical image based PSMs, when assessing PSM credibility, and thereby will contribute to increased reliability and confidence in PSMs across medical specialties and for a wide range of PSM applications, from evaluating medical products to serving as clinical decision-making tools.

## Supporting information

S1 Text Supplementary material. The supplementary material document contains further details on the simulation studies and potential gradations for applying ASME V&V40 with patient specific models.
(PDF)
