# Supplementary material for: Credibility assessment of patient-specific computational modeling using patient-specific cardiac modeling as an exemplar
Source: PLoS Comput Biol. 2022 Oct 10;18(10):e1010541. doi: 10.1371/journal.pcbi.1010541 (PMC9550052; doi:10.1371/journal.pcbi.1010541)
Supplement: S1 Text — The supplementary material document contains further details on the simulation studies and potential gradations for applying ASME V&V40 with patient specific models. (PDF) [file pcbi.1010541.s001.pdf]

Supplementary Material for: Credibility Assessment of Patient-Specific Computational Modeling using Patient-Specific Cardiac Modeling as an Exemplar

Suran Galappaththige[1], Richard A. Gray[1], Caroline Mendonca Costa[2], Steven Niederer[2], Pras Pathmanathan[1,*]
[1]Center for Devices and Radiological Health, US Food and Drug Administration,
[2]School of Biomedical Engineering & Imaging Sciences, King's College London,
[*]corresponding author, pras.pathmanathan@fda.hhs.gov

S1. Simulation study details

In this section we provide additional details for the two studies performed in Section 3.

Mesh resolution study in Section 3.1: The monodomain equation was solved using the ten Tusscher 2004 cell model [S1] for normal myocardium. Following [S2, S3] the same cell model with no modifications was used for border zone as well. Scar was treated as a perfect insulator and implemented by imposing a zero flux boundary condition on the scar boundary [S4]. Tissue conductivity was chosen to be transversely isotropic with values of 1.50 mS/cm in the fiber direction and 0.39 mS/cm in the cross-fiber directions, BZ was isotropic using 0.0767 mS/cm; all values chosen to match [S2]. A spherical region of radius 0.25cm centered at the chosen apical node was stimulated, using a square wave stimulus with magnitude -30000 uA/cm^3 and duration 2ms. Monodomain equation capacitance and surface-area-to-volume ratio were set to 1 uF/cm^2 and 1400/cm respectively.

Uncertainty quantification study in Section 3.2: Governing equations (monodomain), tissue and BZ cell model and treatment of scar was as above, except the TT04 cell model was paced at 2Hz for 1000 cycles in a single cell simulation prior to use in the tissue simulation. Tissue conductivity was chosen to be transversely isotropic with values of 1.54 mS/cm in the fiber direction and 0.29 mS/cm in the cross-fiber directions, with BZ conductivity 0.0768 mS/cm in all directions. There are the exact values used in [S2]. (Note however that our cardiac EP model differs from the original study in [S2]: we solve the monodomain equations with TT04 using the monodomain solver implemented in the C++ computational modeling software Chaste (https://www.cs.ox.ac.uk/chaste/) using a Linux workshop, whereas [S2] solved the reaction-Eikonal equations with TT06 using the reaction-Eikonal solver implemented in Cardiac Arrhythmia Research Package (CARP), also C++ and a Linux workstation [S5]. Therefore, while our baseline simulations reproduce the conclusion of [S2] the absolute varies of HRGV differ from [S2]). Simulations on a three-dimensional slab with a similar mesh resolution to the patient meshes were performed to compute conduction velocity given these conductivities, which was found to be 66cm/s. For the low and high conductivity simulations, tissue conductivities were scaled by constant factors (so that anisotropy ratio was maintained), with factor values chosen so that fiber direction in the slab simulations was 80% of 66cm/s (factor of 0.69 found) and 120% of 66cm/s (factor of 1.41 found).

S2. Potential gradations for applying ASME V&V40 with patient specific models.

In this section we provide the full gradations for some of the credibility factors discussed in Section 4.

Original ASME V&V40 example gradations reprinted from ASME V&V 40-2018, by permission of The American Society of Mechanical Engineers. All rights reserved.

S2.1 Verification credibility factors

Discretization error

See main body

Numerical Solver Error

Example gradation in ASME V&V40	Possible gradation for PSM-CT	Possible gradation for PSM-VC
(a) No solver parameter sensitivity was performed.<br><br>(b) No solver parameter sensitivity was performed. Solver parameters were established based on values from a previously verified computational model.<br><br>(c) Problem-specific sensitivity study was performed on solver parameters, confirming that changes in simulation results due to changes in the solver parameters were negligible relative to the model accuracy goal	(a) No solver parameter sensitivity was performed.<br><br>(b) No solver parameter sensitivity was performed. Solver parameters were established based on values from a previously verified computational model.<br><br>(c) Problem-specific sensitivity study was performed on solver parameters, confirming that changes in simulation results due to changes in the solver parameters were negligible relative to the model accuracy goal, for one or a small number of patients only.<br><br>(d) As (c), except with a range of representative patients.	(a) No solver parameter sensitivity was performed.<br><br>(b) No solver parameter sensitivity was performed. Solver parameters were established based on values from a previously verified computational model.<br><br>(c) Problem-specific sensitivity study was performed on solver parameters, confirming that changes in simulation results due to changes in the solver parameters were negligible relative to the model accuracy goal, for one or a small number of subjects only.<br><br>(d) As (c), except with a range of representative patients.

Use error

Example gradation in ASME V&V40	PSM-CT with no manual stages	PSM-CT with manual stages (e.g., semi-automated image segmentation)	Possible gradation for PSM-VC
(a) Inputs and outputs were not verified.<br><br>(b) Key inputs and outputs were verified by the practitioner.<br><br>(c) Key inputs and outputs were verified by internal peer review.<br><br>(d) Key inputs and outputs were verified by reproducing simulations as part of an external peer review.	Same as original	Split factor into two sub-factors<br><br><u>Use error – objective inputs</u><br>Gradation: same as column 1<br><br><u>Use error – subjectively-chosen inputs</u><br>Example gradation:<br>a) No user variability assessment was performed.<br>b) Inter-user variability was assessed, but not intra-user variability.<br>c) Intra- and inter-user variability was assessed.	Same as original

S2.2 Validation credibility factors related to the model

Model inputs – Sensitivity analysis and uncertainty quantification

The table below provides example gradations for a merged SA/UQ credibility factor, broken down into new sub-factors

**Credibility factor**	**Possible gradation for PSM-CT**	**Possible gradation for PSM-VC**
SA/UQ for model inputs – inputs analyzed	a) No SA/UQ<br>b) Expected key personalized inputs analyzed<br>c) Expected key personalized and non-personalized inputs analyzed<br>d) Wide range of personalized and non-personalized inputs analyzed	Same
SA/UQ for model inputs – rigor of input uncertainty characterization	a) Arbitrary choices, e.g., +/- 10%<br>b) Crude characterization of input uncertainty<br>c) Precise characterization of input uncertainty but correlation between inputs neglected.<br>d) Precise characterization of input uncertainty with correlations characterized	Same
SA/UQ for model inputs – number of patients	a) SA/UQ performed on one patient only<br>b) SA/UQ performed on small number of patients only<br>c) SA/UQ performed on large number of patients covering patient population	a) SA/UQ performed on one patient only<br>b) SA/UQ performed on small number of patients only<br>c) SA/UQ performed using all patients in virtual cohort
SA/UQ for model inputs – output quantities	a) Uncertainty in inputs propagated to compute uncertainty in an output that differs from tool output<br>b) Uncertainty in inputs propagated to compute uncertainty in the tool output	a) Uncertainty in inputs propagated to compute uncertainty in an output that differs from the output analyzed in the simulation study.<br>b) Uncertainty in inputs propagated to compute uncertainty the output analyzed in the simulation study.<br>OR<br>Uncertainty in inputs propagated to assess impact on simulation study conclusion.

S2.3 Validation credibility factors related to the comparator

In the below, original cohort refers to the patient cohort from which the virtual cohort was developed.

ASME V&V40 credibility factor	Example gradation in ASME V&V40	Potential interpretation for some PSM cases	Potential new gradations for some PSM-CT cases	Potential new gradations for some PSM-VC cases
Quantity of Test Samples	(a) A single sample was used.<br>(b) Multiple samples were used, but not enough to be statistically relevant.<br>(c) statistically relevant number of samples were used	Number of validation subjects	(a) Single subject<br>(b) Multiple subjects, not enough to be statistically relevant<br>(c) Statistically relevant number of subjects	(a) Validation not performed for any subject in original cohort<br>(b) Validation performed for some subjects in original cohort<br>(c) Validation performed for all subjects in original cohort
Range of Characteristics of Test Samples	(a) One or more samples with a single set of characteristics were included.<br>(b) Samples representing a range of characteristics near nominal were included.<br>(c) Samples representing the expected extreme values of the parameters were included.<br>(d) Samples representing the entire range of parameters were included.	Range of characteristics of validation subjects	(a) All validation subjects similar<br>(b) Limited range of characteristics in validation subjects<br>(c) Wide range of characteristics in validation subjects	(a) All validation subjects similar<br>(b) Limited range of characteristics in validation subjects<br>(c) Wide range of characteristics in validation subjects
Characteristics of Test Samples	(a) Test samples were not measured and/or characterized.<br>(b) One or more key characteristics of the test samples were measured.<br>(c) All key characteristics of the test samples were measured.	Patient data collected	(a) Key patient data missing *[e.g., because retrospective study]*<br>(b) Most key patient data was obtained.<br>(c) All key patient data was obtained.	(a) Key patient data missing *[e.g., because retrospective study]*<br>(b) Most key patient data was obtained.<br>(c) All key patient data was obtained.
Measurements of Test Samples	(a) Samples were not characterized or were characterized with gross observations, and measurement uncertainty was not addressed.<br>(b) Uncertainty analysis incorporated instrument accuracy only.<br>(c) Uncertainty analysis incorporated instrument accuracy and repeatability (i.e., statistical treatment of repeated measurements).<br>(d) Uncertainty analysis incorporated a comprehensive uncertainty quantification, which included	Patient measurements	(a) Patient measurements were not characterized or were characterized with gross observations, and measurement uncertainty was not addressed.<br>(b) Uncertainty analysis incorporated instrument accuracy only.<br>(c) Uncertainty analysis incorporated instrument accuracy and repeatability (i.e., statistical treatment of repeated measurements).<br>(d) Uncertainty analysis incorporated a comprehensive	(a) Patient measurements were not characterized or were characterized with gross observations, and measurement uncertainty was not addressed.<br>(b) Uncertainty analysis incorporated instrument accuracy only.<br>(c) Uncertainty analysis incorporated instrument accuracy and repeatability (i.e., statistical treatment of repeated measurements).

	instrument accuracy, repeatability, and other conditions affecting the measurements.		uncertainty quantification, which included instrument accuracy, repeatability, and other conditions affecting the measurements.	(d) Uncertainty analysis incorporated a comprehensive uncertainty quantification, which included instrument accuracy, repeatability, and other conditions affecting the measurements.

References

S1. ten Tusscher, K.H., et al., *A model for human ventricular tissue.* Am J Physiol Heart Circ Physiol, 2004. **286**(4): p. H1573-89.

S2. Costa, C.M., et al., *Pacing in proximity to scar during cardiac resynchronization therapy increases local dispersion of repolarization and susceptibility to ventricular arrhythmogenesis.* Heart rhythm, 2019. **16**(10): p. 1475-1483.

S3. Mendonca Costa, C., et al., *Modeling the electrophysiological properties of the infarct border zone.* Frontiers in physiology, 2018. **9**: p. 356.

S4. Connolly, A.J. and M.J. Bishop, *Computational representations of myocardial infarct scars and implications for arrhythmogenesis.* Clinical Medicine Insights: Cardiology, 2016. **10**: p. CMC. S39708.

S5. Neic, A., et al., *Efficient computation of electrograms and ECGs in human whole heart simulations using a reaction-eikonal model.* Journal of computational physics, 2017. **346**: p. 191-211.
